# The Impact of Glycemic Status and Metformin Administration on Red Blood Cell Indices and Oxidative Stress in Type 2 Diabetic Patients

**DOI:** 10.21315/mjms2019.26.4.6

**Published:** 2019-08-29

**Authors:** Adel Abdel-Moneim, Eman Salah Abdel-Reheim, Margit Semmler, Wessam Addaleel

**Affiliations:** 1 Physiology Division, Zoology Department, Faculty of Science, Beni-Suef University, Egypt; 2 Diabetes Research Institute, Düsseldorf University, Düsseldorf, Germany

**Keywords:** red blood cell indices, glycemic status, metformin, oxidative stress

## Abstract

**Background:**

Most guidelines all over the world recommended metformin as the first-line treatment for in type 2 diabetic patients. Therefore, the present study was suggested to assess the outcome of metformin administration and glycemic status on alterations in red blood cell (RBCs) indices as well as the oxidative stress in type 2 diabetic patients.

**Methods:**

Between December 2016 and October of 2017, a total of 158 eligible individuals were classified as 50 healthy subjects and 108 diabetic patients who were subdivided into six groups according to the type of anti-diabetic treatments.

**Results:**

Overall, the results elucidated that hemoglobin concentration was markedly diminished, while red cell distribution width (RDW) value was significantly (*P* < 0.001) elevated in all diabetic groups as compared to control. Moreover, in all diabetic groups, malondialdehyde (MDA) concentration was elevated noticeably (*P* < 0.001), while reduced glutathione (GSH) revealed a lower concentration (*P* < 0.001) than that of control.

**Conclusion:**

The present study exhibited the amelioration effect of metformin administration on oxidative stress and glycemic status which reflected on some RBCs indices. However, hemoglobin concentration showed a noticeable diminution in all metformin-treated groups in spite of the improvement in glycemic and oxidative stress status which indicated that the metformin-induced anemia is independently from diabetic complications.

## Introduction

Diabetes is a worldwide health problem with a close link exists between diabetes and cardiovascular disease (CVD), whereas CVD is the most prevalent cause of mortality and morbidity among the diabetic populations (1). The prevalence of type 2 diabetes mellitus (T2DM) has been globally growing rapidly. Approximately 592 million people worldwide will become diabetic by the year 2035, with a universal prevalence of 10.1% (2).

Currently, the orally employed antidiabetic agents: biguanides (e.g., metformin), sulfonylureas (e.g., glimepiride), α-glycosidase inhibitors (e.g., acarbose) and thiazolidinediones (e.g., pioglitazone), act to regulate a specific pathological pathway (3). Metformin has been utilised for 50 years and approved by the US Food and Drug Administration (FDA) in 1994. Moreover, most guidelines all over the world have recommended metformin as the first-line treatment for T2DM patients (4). Glimepiride is a second-generation sulfonylurea that stimulates pancreatic β-cells to release insulin and it has been shown to act through additional extrapancreatic mechanisms. In addition, metformin is preferentially selected for combination therapy with sulfonylurea or insulin to achieve the glycemic target in patients who are not satisfactorily controlled by monotherapy alone (5).

Oxidative stress and inflammation contribute to the development of diabetic complications. Intracellular hyperglycemia promotes the production of mitochondrial reactive oxygen species (ROS). ROS is directly increase the expression of inflammatory and adhesion factors, the formation of oxidised-low density lipoprotein, and insulin resistance (6). Depletion of blood glutathione (GSH) has been recorded in many clinical issues including T2DM (7). Kumawat et al. revealed that hyperglycemia induces oxidative stress through increasing the malondialdehyde (MDA) levels in diabetic patients (8). Moreover, oxidative stress produced from an imbalance between free radicals and the body’s antioxidant defense systems leads to red blood cell dysfunction and tissue injury (9).

Recently, a great attention re-given to the hematological indices as predictors of endothelial dysfunction and inflammation status (10). Anemia is twice in diabetic patients, and mild anemia also has been recorded in diabetics with normal kidney function (11). In addition, anemia is associated with duration of disease and microvascular complications; diabetic neuropathy, nephropathy, retinopathy and CVD (12). Red blood cells (RBCs) count is a pivotal marker for the ability to recognise diabetic patients at risk of microvascular complications. Lower RBCs counts are, therefore, considered as an independent predictor biomarker of the risk of microvascular complications in T2DM patients (13). Additionally, erythrocytes of diabetic patients aggregate more readily that is obviously enhance whole-blood viscosity (WBV), and adversely influence the microcirculation and finely leading to microangiopathy (14).

Among RBCs indices, the red blood cell distribution width (RDW) is a simple and non-expensive parameter, which can reflect the level of erythrocyte volume heterogeneity (anisocytosis) and is commonly used in the laboratory for differential analysis of anemia (15). Furthermore, RDW values showed a significant increase in T2DM patients than in control subjects (16). Thus, the ideal therapy for diabetes would be a medication that besides its hypoglycemic effect can reduce the oxidation status and diabetic complications. Consequently, the goal of our study was to evaluate the impact of metformin administration (mono- and dual therapy) and glycemic control on RBC indices alterations and oxidative stress status in Egyptian patients with T2DM.

## Subjects and Methods

### Patients Inclusion and Exclusion Criteria

The study was conducted on 158 individuals of both sexes (83 males and 75 females), aged between 30 and 75 years. These individuals enrolled in this study were classified into 50 normal healthy subjects and 108 T2DM patients who were followed up at the Diabetic Section of General Institution of Healthy Insurance, Egypt, between December 2016 to October 2017. Written agreements were obtained from all patients before participation in the study. The present study was performed in accordance with the declaration of Helsinki and good clinical practice guidelines and also approved by the committee of General Institutions of Health Insurance.

Enrolled patients were allocated to normal healthy subjects (control) who had no previous history of chronic diseases and free of type I or 2 diabetes, and 108 patients diagnosed as T2DM according to WHO 1999 criteria. Pregnant and lactating women, patients receiving immuno-modulatory drugs and patients with medical conditions such as infections, cerebrovascular diseases, ischemic heart disease, malignancies, autoimmune disorders, eczema, respiratory disorder, thyroid dysfunction, kidney failure, liver dysfunction and alcohol abuse were excluded from the study. In addition, diabetic patients who underwent medication changes during the 2 months preceding participation were also excluded.

### Study Design

The diabetic patients were subdivided into six groups according to the treatment administration.

**Table:** 

1. Group 1: normal healthy subjects (control)	(50 subjects)
2. Group 2: diabetics (recent diagnoses) nontreated	(20 patients)
3. Group 3: diabetics treated with metformin only	(15 patients)
4. Group 4: diabetics treated with glimepiride only	(19 patients)
5. Group 5: diabetics treated with metformin + glimepiride	(15 patients)
6. Group 6: diabetics treated with insulin only	(20 patients)
7. Group 7: diabetics treated with metformin + insulin	(19 patients)

The demographic data regarding anthropometric variables such as height, weight, body mass index (BMI), gender, duration of disease and blood pressure (BP) were collected. Blood samples were taken from participants after overnight fasting in EDTA and plain tubes (4 mL each). Following an incubation period of 30 min at room temperature, blood in plain tubes was centrifuged at 4,000× g for serum isolation. Sera were rapidly separated, aliquoted and stored at −40 °C until the biochemical measurements. The second tube blood sample was taken on potassium fluoride for immediate glucose estimation. The third part of the blood sample was taken on EDTA for determination of HbA1c levels and complete blood picture at the same time, then, the blood was centrifuged, the plasma and leucocytes layer were then removed and the packed erythrocyte sediments were washed three times with normal saline and hemolyzed by adding approximately 1.5 volumes of ice-cold distilled water. The stock hemolysate stored at −40 °C for estimation of MDA and GSH levels. Fresh morning urine samples were obtained for the measurement of microalbuminuria concentration.

### Laboratory Assays

The concentrations of glucose, creatinine, total cholesterol, triglyceride and HDL-c were estimated using commercially available assay kits obtained from Reactivos Spinrect, Spain. low density lipoprotein (LDL)-cholesterol level was calculated according to Friendewald formula (17). The anti-atherogenic factor indices were calculated using Ross formula (18). HbA1c % was determined using reagent kits purchased from Spectrum. Microalbuminuria in the urine was estimated by reagent kits purchased from Bio-Diagnostic. Moreover, reduced glutathione (GSH) and lipid peroxidation (determined indirectly by measuring MDA) were assayed in the blood hemolysate using colorimetric kits (BioVision, Milpitas, CA, USA) according to the kit instructions provided. Hematology profile; hemoglobin (Hb), hematocrit (PCV), mean corpuscular volume (MCV), mean corpuscular hemoglobin (MCH), mean corpuscular hemoglobin concentration (MCHC), RDW values and RBCs count were determined using a MICROS ABX auto-analyser according to the manufacturer’s protocol.

### Statistical Analysis

The data were analysed by one-way analysis of variance (ANOVA). To compare the difference between the groups, post hoc testing was performed by the Duncan test with least significant difference (LSD). Pearson’s correlation coefficient analysis was used to determine the correlations between different studied parameters. Statistical analysis was performed using the Statistical Package for the Social Science (SPSS) for Windows (version 22.0, Chicago, IL, USA). Data are expressed as mean (SD). Values with *P* < 0.05 were considered statistically significant.

## Results

**Figure 1:**
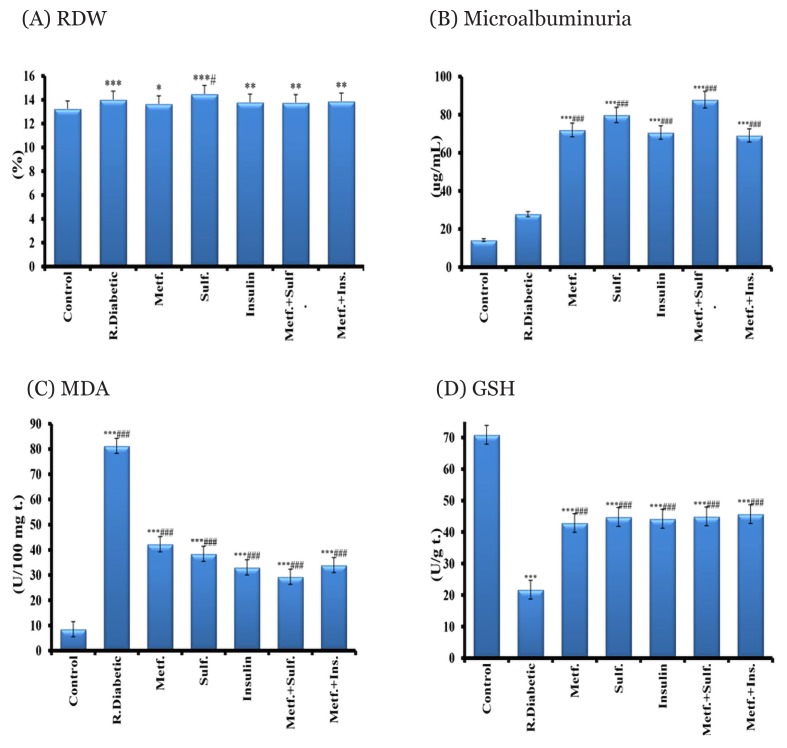
Values of RDW% (A), microalbuminuria (B), MDA (C) and GSH (D) of control, recent diabetic and different metformin treated groups ^*^Significance compared to normal control, ^*^P < 0.05, ^**^P < 0.01, ^***^P < 0.001, ^#^significance compared to the recent diabetic. ^#^*P* < 0.05, ^##^*P* < 0.01, ^###^*P* < 0.001. Metf = metformin, R diabetic = recent diabetic, Sulf = sulfonylurea (glimepiride), Ins = insulin,

The current results revealed that family history, BMI, total cholesterol, LDL-c, and HbA1c levels were significant elevated in recently diagnosed diabetic patients as well as in all treated diabetic groups compared to the healthy control (Table 1). In addition, MCV, MCH and MCHC values showed non-significant changes in all studied groups compared to healthy control. A non-significant lowering in RBCs count was found in all diabetic groups except patients treated with metformin plus insulin [4.89(0.7) vis 4.30(0.5)]. In addition, HCT% revealed a significant decrease in diabetic patients treated with glimepiride therapies compared to the healthy control group. Moreover, Hb concentration was reduced markedly in different diabetic groups, while RDW values had elevated significantly (*P* < 0.001) in all diabetic groups as compared to the healthy control [Figure 1(A)]. While, urinary microalbuminuria was significantly elevated (*P* < 0.001) in the treated diabetic groups when compared to the healthy and recent-diabetic groups [Figure 1(B)]. However, lipid peroxidation biomarker (MDA) was elevated markedly (*P* < 0.001) in all diabetic groups, while the antioxidant GSH level was lowered noticeably (*P* < 0.001) in different diabetic groups when compared to the healthy control ones [Figures 1(C) and 1(D)].

**Figure 2:**
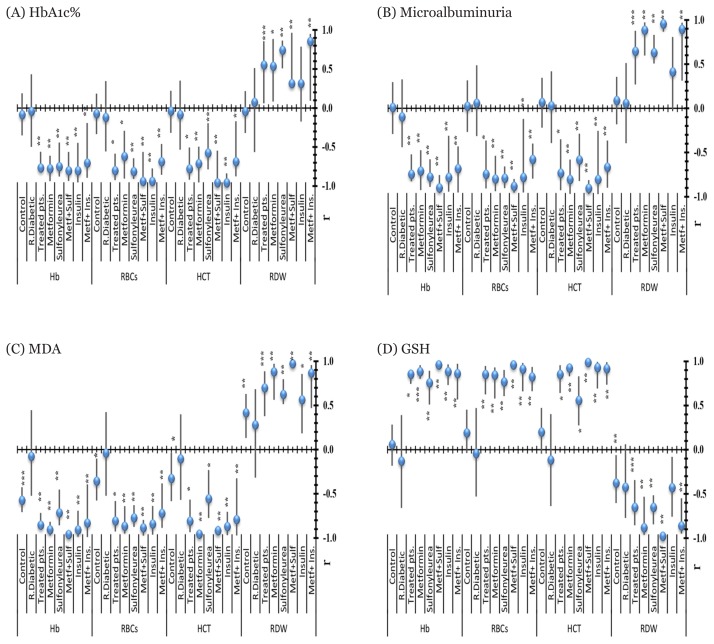
Correlation coefficient of Hb, RBCs, HCT and RDW values with HbA1c% (A), microalbuminuria (B), MDA (C) and GSH (D) *Correlation is significant at the 0.05 level. **Correlation is significant at the 0.01 level. ***Correlation is significant at the 0.001 level. R = recent, Metf = metformin, R. diabetic = recent diabetic, Sulf = sulfonylurea (glimepiride), Ins = insulin, HbA1c = glycosylated hemoglobin, r = correlation coefficient

**Figure 3:**
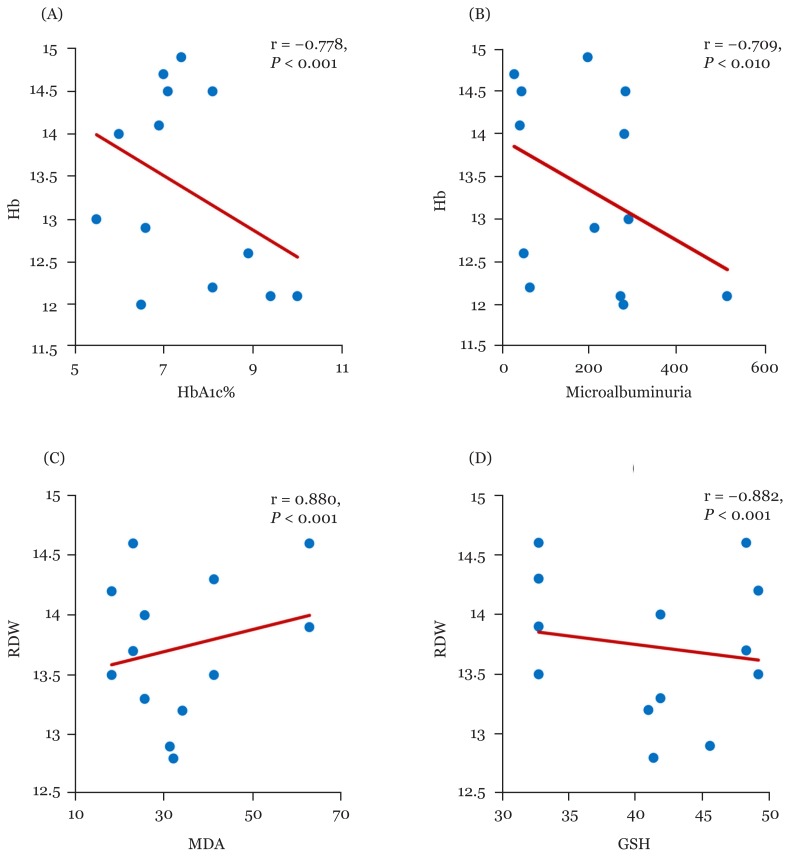
Correlations of Hg% with HbA1c% (A) and microalbuminuria (B), and RDW with MDA (C) and GSH (D) among metformin monotherapy group HbA1c = glycosylated hemoglobin, r = correlation coefficient

**Figure 4:**
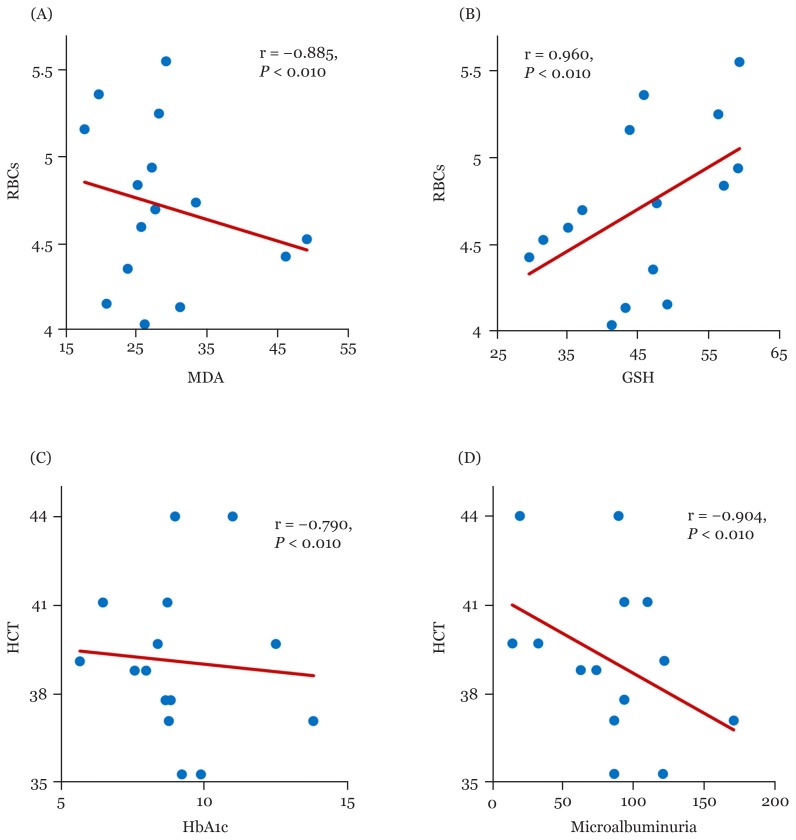
Correlations between RBCs with MDA (A) and GSH (B), and HCT with HbA1c (C) and microalbuminuria (D) among metformin plus glimepiride group HbA1c = glycosylated hemoglobin, r = correlation coefficient

**Figure 5:**
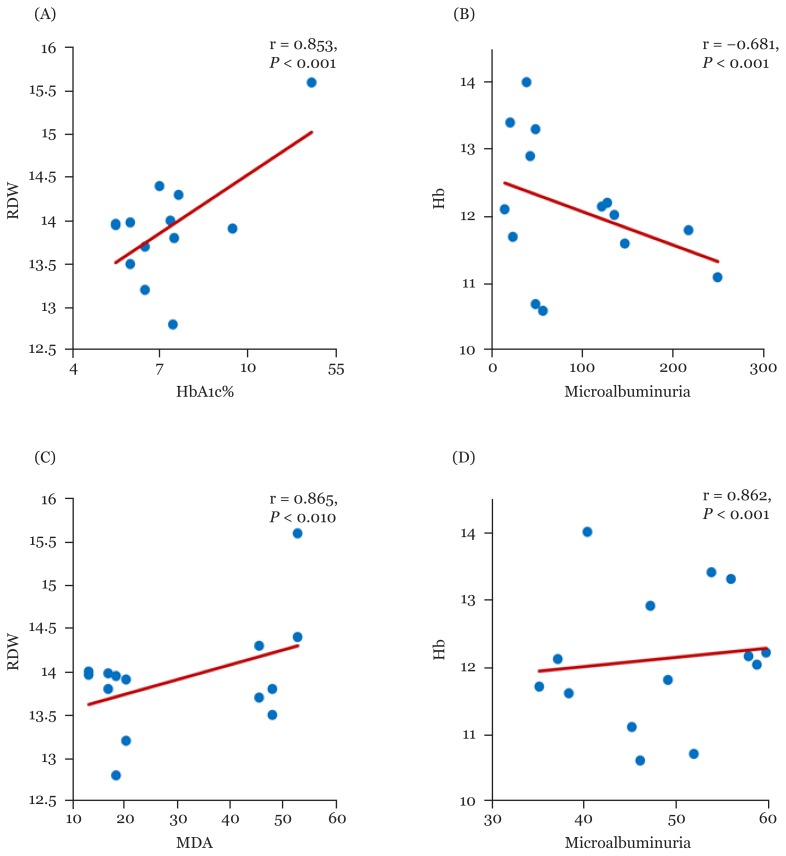
Correlations of RDW with HbA1c% (A) and MDA (C), and Hb% with microalbuminuria (B), and GSH (D) among metformin plus insulin patients HbA1c = glycosylated hemoglobin, r = correlation coefficient

Overall, Hb showed a negative significant correlation with HbA1c, microalbuminuria, and MDA in patients treated with metformin monotherapy [r; −0.778 (95%CI: −0.937, −0.612), r; −0.709 (95% CI: −0.925, −0.475), r; −0.906 (95% CI: −0.971, −0.818), respectively] and dual therapy groups, however, HB observed a positive significant correlation with GSH in all metformin therapy groups [Table 2, Figures 2(A), 2(B), 3(A), 3(B), 5(B) and 5(D)]. Moreover, RBCs count revealed a significant negative correlation with HbA1c, MDA and microalbuminuria in metformin therapies groups. While, RBCs count induced a positive significant correlation with GSH in metformin monotherapy as well as combination therapy groups with a sulphonylurea (glimepiride) and insulin (r; 0.960 (95% CI: 0.935, 0.982, 0.824(95% CI: 0.661, 0.936), respectively) [Table 2, Figures 2(A), 2(B), 4(A) and 4(B)].

Moreover, HCT value observed a negative correlation between HbA1c, microalbuminuria and MDA with metformin monotherapy and combined therapies of metformin. On the other hand, HCT value had a positive significant correlation between GSH with mono-and dual metformin therapies with a sulphonylurea and insulin [0.925 (95% CI: 0.831, 0.975), 0.988 (95% CI: 0.974, 0.997) 0.918 (95% CI: 0.735, 0.991), respectively] [Table 2, Figures 2(C), 2(D), 4(C) and 4(D)]. RDW level showed a significant positive correlation between HbA1c, MDA and microalbuminuria with metformin monotherapy (r; 0.536 (95% CI: 0.082, 0.885), 0.885 (95%CI: 0.596, 0.973), 0.880 (95%CI: 0.563, 0.971), respectively) and dual therapies, while, it correlated negatively with GSH in all tested groups [Table 2, Figures 2(C), 2(D), 3(C), 3(D), 5(A) and 5(C)].

## Discussion

Overall, our results confirmed that Hb concentration was lowered in all diabetic groups, while RDW was elevated significantly in diabetic patients. Also, the erythrocytes (RBCs) count revealed a significant decrease in patients treated with metformin plus insulin. The decrease in RBCs counts in diabetic patients may be due to RBC membrane protein alterations, a decrease in hemoglobin levels and erythropoietin deficiency (13). Hemoglobin is the major component of erythrocytes; thus, when the HbA1c level is elevated, hyperglycemia may increase the β-sheet structure content of Hb causing it to aggregate which subsequently increases WBV (19). In fact, the excessive aggregation of RBC is one of the most prominent features in patients with diabetes with poor glycemic control. The erythrocyte aggregation is an important hemorheological parameter because it directly affects WBV (20). Also, some recent epidemiological studies have reported that T2DM is characterised by increased erythrocyte osmotic fragility (21). In diabetes, erythrocyte membranes are affected by the chronic exposure to glucose, and several biochemical modifications are triggered, with subsequent structural and functional disruption of erythrocytes and decreased the lifespan of RBCs (22) which may be related to RBCs count dimension. Meanwhile, RBC properties are critically affected by hyperglycemia and decreased deformability (23).

Anemia is a highly prevalent condition in people with T2DM. The causes of diabetic anemia are multifactorial including inflammation, concomitant autoimmune diseases, antidiabetic medication, hormonal changes and kidney diseases (12). The manifestation of anemia in diabetics has been attributed to the increase of non-enzymatic glycosylation of RBC membrane proteins, which was correlated with hyperglycemia (24). The protein oxidation and hyperglycemia in diabetics induced an elevation in the production of lipid peroxides that may lead to RBCs hemolysis (25) and subsequently decrease in RBCs count and hemoglobin levels. Moreover, the diabetic and anemic patients had high levels of C-reactive protein and ferritin ultra-sensible with low iron contents which might refer to association between ferritin increase and chronic inflammatory process in diabetics (26). However, long-term treatment with metformin is known to be associated with vitamin B12 deficiency and anemia in patients with T2DM (27, 28). It is reported that long-term use of metformin was associated with biochemical B12 deficiency and anemia (28). Since vitamin B12 is essential to nutrition, the metformin-induced B12 reduction may have detrimental effects in patients with T2DM (29). Moreover, Kang et al. recommended the need for regular vitamin B12 monitoring in patients with T2DM, particularly patients receiving a higher daily dosage of sulfonylurea plus metformin treatment for a long time period (27).

The main pathophysiological consequences of free radical-induced lipid peroxidation are disturbing the assembly of cell membranes, which inevitably will impact membrane fluidity, and lipid-protein interaction dynamics, membrane permeability, and physicochemical properties (30). Moreover, in this study, MDA concentration was elevated markedly in all diabetic groups, while, the GSH level was lowered significantly in diabetic subjects. In addition, RBCs count, Hb and HCT values showed a negative correlation between HbA1c, MDA and microalbuminuria with metformin therapies. However, a positive correlation with GSH in all diabetic groups was observed. Hyperlipidemia and increased lipid peroxidation were strongly associated with increased systemic inflammation, and as well as the increased MDA production has also been recorded in the erythrocyte membrane of diabetic patients (8). Additionally, in parallel with our findings, De Souza Bastos et al. suggested that diabetes was associated with dyslipidemia and increased lipid peroxidation (31). Moreover, Lee et al. concluded that the high level of lipids may alter the morphology and flow behaviour of RBCs, which could contribute to the impairment of microcirculatory-related disorders (32).

In addition, in accordance with the current study, several findings showed that patients with T2DM had lower GSH content in erythrocytes than that in the control group. Also, a decrease in intracellular glutathione level in patients with T2DM was reported especially in the presence of microvascular complications (33), which was depend on the degree of hyperglycemia. Furthermore, patients with T2DM have a lower concentration of intracellular GSH, which increase the susceptibility of blood cells to the damaging effects of ROS. Waggiallah and Alzohairy found a significant impact of oxidative stress (low glutathione) on glutathione peroxidase which could reduce Hb concentration in diabetic patients, which means that oxidative stress of diabetes is one of the causes of anemia in diabetics independently from diabetic nephropathy (34).

In accordance with our results, the RDW values were significantly higher in diabetic patients than that of healthy subjects (35). Furthermore, the RDW level showed a positive correlation with HbA1c, MDA and microalbuminuria in all metformin administered therapies. The exact pathophysiological mechanism underlying the association between RDW and diabetes still unknown, however, it was established that inflammation and oxidative stress can alter erythrocyte homeostasis and increase RDW values. Although the specific mechanisms between the RDW and adverse health outcomes were fully unclear, it was suggested that it could be related to increased oxidative stress and inflammatory cytokines (36). Inflammation inhibits bone marrow function and iron metabolism, and pro-inflammatory cytokines have been proven to inhibit erythropoietin-induced maturation and proliferation of erythrocytes. All these factors may contribute to the increase in RDW% (37). Additionally, high RDW indicated impairment of erythropoiesis, reflecting chronic inflammation and increased level of oxidative stress, both of which were significant signs of T2DM (38). As we all know, diabetes mellitus is considered as a chronic inflammatory disease (39). Thus, the achievement of the glycemic remission might result from a lower level of inflammation and oxidative stress which was indicated by lowering the RDW level. Increased RDW value was therefore reflect the significant deregulation of erythrocyte homeostasis. This deregulation induced chronic inflammation, the elevation of oxidative stress, erythrocyte fragmentation, poor nutritional status, hypertension, dyslipidemia, impairment of erythropoiesis, and erythropoietin dysfunction (15).

Microalbuminuria, the best sign for diabetic nephropathy development in T2DM, was also, accepted as an indicator of diabetic microangiopathy (40). Urinary microalbuminuria, in this study, was significantly increased in all treated diabetic groups. The erythropoietin dysregulation, caused by early damage to renal tubules, has been suggested as one of the contributors to anemia in patients with diabetes (41). Magri and Fava found that RDW was strongly associated with diabetic nephropathy and may be independently associated with microalbuminuria in patients with T2DM (42). Some investigators attributed the association between RDW and microalbuminuria to the presence of chronic inflammation in diabetics (43).

When fructose-fed rats were treated with metformin, the imbalance between peroxidation and antioxidants defense system was mitigated (44). However, treatment with glimepiride and vildagliptin improved erythrocyte deformability in patients with T2DM, the improvement seemed to be correlated with improved glycemic control (45). Moreover, it was observed that patients of good glycemic control have lower RDW than the patients of poor control (35). On the other hand, metformin or glibenclamide might ameliorate oxidative stress in the kidneys of diabetic rats to a certain extent with regard to SOD and MDA (46). Tessier et al. reported that the improved glycemic control with gliclazide and metformin therapy was associated with improvement in the antioxidant/lipid peroxidation status (47). In addition, Chukwunonso-Obi et al. found that administration of metformin, glibenclamide, and repaglinide exhibited a significant reduction in MDA concentration and considerable improvement in the altered activities of antioxidant enzymes (48).

## Conclusion

The data indicated that metformin administration can induce amelioration in hyperglycemia and oxidative stress as well as inflammation status regarded to MDA, GSH and RDW levels. However, Hb concentration showed a reduction in metformin-treated groups in spite of improvements in glycemic and oxidative status. This mean that the metformin-induced anemia is independent of diabetes.

## Figures and Tables

**Table 1:** Demographic, biochemical and RBC indices in control, recent diabetic and treated diabetic groups

*N* =	Control 50	R Diabetic 20	Metformin 15	Glimepiride 19	Metf +Sulf 15	Insulin 20	Metf+Ins 19
**Gender**							
*N*(%)							
M ♂	25 (50)^a^	10 (50)^c^	8 (53) ^c^	9 (47)^c^	8 (53)^c^	10(52)^c^	13(65)^b^
F ♀	25 (50)^a^	10 (50)^b^	7(47)^c^	10(53)^b^	7(47)^c^	9(47)^b^	7(35)^c^
F. hist.	(0)^a^	4(20)^b^	6 (40)^bc^	5(26)^b^	4(27)^b^	11(58)^d^	8(40)^c^
Duration (y) M(SD)	0.0(0) ^a^	0.0(0)^a^	4.4(3) ^b^	5.8(4)^b^	6.2(5)^b^	16(8)^c^	14(7) ^c^
Age (y)	42(16) ^a^	49(14)^a^	57(8) ^bc^	63(11) ^c^	55(9) ^bc^	57(8)^bc^	62(7) ^c^
BMI	27(7) ^a^	36(5)^b^	33(7) ^ab^	34(4)^ab^	32(8) ^ab^	33(10) ^ab^	29(4) ^a^
HbA1c%	5.11(0.51)^a^	9.78(1.46)^d^	7.31(1.32)^b^	7.55(1.19)^bc^	9.80(2.59)^d^	7.58(1.94)^bc^	8.51(2.18)^c^
Creatinine (mg/dL)	1.03(0.23)^abc^	0.96(0.25) ^abc^	0.83(0.18) ^a^	1.15(0.45) ^bc^	1.04(0.25) ^abc^	1.22(0.89)^c^	0.90(0.24)^ab^
TC (mg/dL)	168(28)^a^	218(48)^bcd^	191(51)^ab^	197(55)^bc^	227(41)^d^	194(24)^abc^	220(42)^cd^
LDL (mg/dL)	99(38) ^a^	151(45) ^bc^	124(49) ^ab^	136(45) ^bc^	165(39) ^d^	128(23) ^bc^	154(39) ^cd^
Hb (g/dL)	14.30(1.0) ^c^	12.27(1.9) ^a^	13.22(0.76)^b^	12.65(1.3) ^a^	12.71(0.5)^a^	12.97(1.3) ^a^	12.14(1.1) ^a^
RBCs (×10^6^/mm^3^)	4.89(0.7)^bc^	4.56(0.7)^ab^	4.73(0.3)^bc^	4.50(0.4)^ab^	4.55(0.4)^ab^	4.74(0.5) ^bc^	4.30(0.5) ^a^
HCT (%)	40.53(3) ^b^	37.36(6) ^ab^	39.18(2) ^ab^	36.65(2) ^a^	38.49(2) ^ab^	39.20(5) ^ab^	37.13(2) ^ab^
MCV (fl)	83.27(2)^ab^	81.98(7) ^a^	82.88(5) ^ab^	82.14(8) ^ab^	85.05(4)^b^	82.65(6) ^ab^	87.15(7) ^b^
MCH (Pg)	28.15(2)	27.08(2)	27.25(2)	27.28(3)	28.18(2)	27.48(2)	28.25(3)
MCHC (g/dL)	33.85(2) ^b^	32.90(2) ^ab^	31.81(3) ^a^	33.16(1) ^b^	33.07(1) ^b^	32.86(1) ^ab^	32.72(1) ^ab^

Data are expressed as mean = SD. Values which share the same superscript symbol are not significantly different. R = recent, Metf = metformin, Sulf = sulfonylurea (glimepiride), Ins = insulin, F hist = family history, BMI = body mass index, HbA1c = glycosylated hemoglobin, TC = total cholesterol, LDL = low density lipoprotein, MCV = mean corpuscular volume, MCH = mean corpuscular hemoglobin, MCHC = mean corpuscular hemoglobin concentration

**Table 2:** Pearson correlation of HbA1c, MDA, GSH and microalbuminuria with RBCs indices in different treated groups

	HbA1c		Microalbuminuria		MDA		GSH	
			
	r	95% CI	r	95% CI	r	95% CI	r	95% CI
Metf								
Hb	−0.778**	(−0.937, −0.612)	−0.709**	(−0.925, −0.475)	−0.906**	(−0.971, −0.818)	0.884***	(0.802, 0.957)
RBCs	−0.621*	(−0.905, −0.291)	−0.798**	(−0.937, −0.537)	−0.867**	(−0.951, −0.640)	0.846**	(0.579, 0.932)
HCT	−0.713**	(−0.953, −0.496)	−0.803**	(−0.968, −0.580)	−0.957**	(−0.987, −0.914)	0.925**	(0.831, 0.975)
RDW	0.536*	(0.082, 0.885)	0.885**	(0.596, 0.973)	0.880**	(0.563, 0.971)	−0.882**	(−0.962, −0.532)
Metf + Sulf								
Hb	−0.838**	(−0.934, −0.562)	−0.899**	(−0.959, −0.754)	−0.961**	(−0.985, −0.917)	0.961**	(0.906, 0.990)
RBCs	−0.774**	(−0.909, −0.563)	−0.882**	(−0.954, −0.799)	−0.885**	(−0.973, −0.795)	0.960**	(0.935, 0.982)
HCT	−0.790**	(−0.926, −0.547)	−0.904**	(−0.970, −0.812)	−0.917**	(−0.971, −0.862)	0.988**	(0.974, 0.997)
RDW	0.888**	(0.630, 0.965)	0.955**	(0.867, 0.986)	0.972**	(0.937, 0.991)	−0.978**	(−0.991, −0.961)
Metf + Ins								
Hb	−0.704**	(−0.192, −0.894)	−0.681**	(−0.431, −0.953)	−0.831**	(−0.394, −0.989)	0.862**	(0.973, 0.568)
RBCs	−0.687**	(−0.855, −0.460)	−0.578**	(−0.795, −0.398)	−0.721**	(−0.885, −0.382)	0.824**	(0.661, 0.936)
HCT	−0.688**	(−0.875, −0.170)	−0.665**	(−0.898, −0.364)	−0.791**	(−0.972, −0.325)	0.918**	(0.735, 0.991)
RDW	0.853**	(0.092, 0.950)	0.895**	(0.313, 0.968)	0.865**	(0.469, 0.957)	−0.862**	(−0.949, −0.549)

* Correlation is significant at the 0.05 level.

** Correlation is significant at the 0.01 level.

*** Correlation is significant

Metf = metformin, Sulf = sulfonylurea (glimepiride), Ins = insulin, HbA1c = glycosylated hemoglobin
